# Cost-effectiveness of a hypothetical cell or gene therapy cure for sickle cell disease

**DOI:** 10.1038/s41598-021-90405-1

**Published:** 2021-05-25

**Authors:** Jonathan Salcedo, Jenniffer Bulovic, Colin M. Young

**Affiliations:** 1 Department of Pharmaceutical and Health Economics, School of Pharmacy, University of Southern California, 635 Downey Way, Verna & Peter Dauterive Hall, VPD 312, Los Angeles, CA 90089-3333 USA; 2 Center for Biomedical Innovation, Massachusetts Institute of Technology, Cambridge, MA USA

**Keywords:** Health care economics, Health services

## Abstract

Sickle cell disease (SCD) is a group of inherited genetic conditions associated with lifelong complications and increased healthcare resource utilization. Standard treatment for SCD in the US varies based on stage of the disease and observed clinical severity. In this study, we aim to evaluate the potential cost-effectiveness of a durable cell or gene therapy cure for sickle cell disease from the US healthcare sector perspective. We developed a lifetime Markov model to evaluate the cost-effectiveness of a hypothetical single-administration durable treatment (DT) for SCD provided at birth, relative to standard of care (SOC). We informed model inputs including direct healthcare costs, health state utility weights, transition probabilities, and mortality rates using a retrospective database analysis of commercially insured individuals and the medical literature. Our primary outcome of interest was the incremental cost-effectiveness ratio (ICER) of DT versus SOC evaluated at a base case willingness-to-pay (WTP) threshold of $150,000 per quality-adjusted life year (QALY). We tested the robustness of our base case findings through scenario, deterministic sensitivity (DSA), and probabilistic sensitivity analyses (PSA). In the base case analysis, treatment with DT was cost-effective with an ICER of $140,877/QALY relative to SOC for a hypothetical cohort involving 47% females. Both males (ICER of $135,574/QALY) and females (ICER of $146,511/QALY) were similarly cost-effective to treat. In univariate DSA the base case ICER was most sensitive to the costs of treating males, DT treatment cost, and the discount rate. In PSA, DT was cost-effective in 32.7%, 66.0%, and 92.6% of 10,000 simulations at WTP values of $100,000, $150,000, and $200,000 per QALY, respectively. A scenario analysis showed cost-effectiveness of DT is highly contingent on assumed lifetime durability of the cure. A hypothetical cell or gene therapy cure for SCD is likely to be cost-effective from the US healthcare sector perspective. Large upfront costs of a single administration cure are offset by significant downstream gains in health for patients treated early in life. We find cost-effectiveness outcomes do not vary substantially by gender; however, several model parameters including assumed durability and upfront cost of DT are likely to influence cost-effectiveness findings.

## Introduction

Sickle cell disease (SCD) is an inherited group of disorders characterized by the presence of hemoglobin S. Individuals inherit the disease from parents that carry the trait or may even exhibit the disease. The clinical manifestation of SCD involves damaged and abnormally shaped red blood cells which result in hemolytic anemia, small blood vessel obstruction, and other resulting complications^1–3^. Informally, sickle cell anemia (SCA) is often used interchangeably with SCD. SCD is associated with reduced life expectancy, significant quality of life detriments, and increased healthcare resource utilization^4^. Severe pain episodes are common in many patients and often require costly hospitalization^5,6^. There are an estimated 100,000 individuals currently living with SCD in the United States^7^.

Current treatment paradigms for SCD in the US vary by age group and generally involve both palliative and preventive care. Adverse events include painful and resource-intensive crises and other acute and chronic complications. Common interventions for these adverse events supported by evidence include vaccination, penicillin, hydroxyurea, blood transfusion, and opioids^5^. Hydroxyurea is typically well-tolerated and effective in decreasing the frequency of pain episodes and acute complications in children and adults with SCD^8,9^. Despite this, utilization in SCD is low, with one estimate finding that only 30% of eligible patients receive it^8,10^. Underuse of hydroxyurea in SCD may be due to patient and provider factors including concerns towards long-term risks.

Few disease modifying therapies are available. Hematopoietic stem cell transplant (HSCT) is a potential curative treatment usually reserved for those with severe progressive disease despite optimal medical management. However, HSCT is not widely available due to substantial barriers to treatment, possible transplant rejection, and various long-term adverse outcomes^11,12^. Two recent treatments voxelotor and crizanlizumab have been shown in clinical trials to exhibit superior hemoglobin (Hb) response rate over placebo and reduce the frequency of vaso-occlusive crises (VOC) over placebo, respectively^13,14^. These trials were cited as evidence in US Food and Drug Administration (FDA) approvals for each of these interventions^13–16^.

In addition to an increasing number of treatments for chronic management of disease-related symptoms, a gene therapy cure aimed at helping patients with SCD produce normal red blood cells has shown promise, and may reach US markets within years^17^. With this comes the potential to treat patients with SCD with a single or short-term administration therapy and avoid disease progression for up to a lifetime, resulting in downstream improvements in life expectancy, quality of life, and treatment cost. The cost-effectiveness of a hypothetical cure for SCD from the US healthcare sector perspective is unknown. In this study we estimate the cost-effectiveness of a hypothetical treatment for SCD provided at birth with varying durability (ranging from 10 years to a lifetime) free of the disease and its related complications.

## Methods

### Study design

We built a Markov model and analyzed it using a cohort to conduct a cost-effectiveness analysis of a hypothetical durable treatment for sickle cell disease relative to standard of care (SOC). The model horizon was lifetime in yearly cycles with a 3% yearly discount on costs and health outcomes. We conducted the analysis from the US healthcare sector perspective. Costs included direct medical costs incurred by patients and providers and our measure of health utility was quality-adjusted life years (QALYs). Our primary outcome of interest was the incremental cost-effectiveness ratio (ICER) between durable therapy and SOC. We evaluated this outcome against a willingness-to-pay (WTP) threshold of $150,000 per QALY in 2018 US dollars. An ICER below our threshold means the intervention is cost-effective^18^. We tested robustness of our base case through univariate, probabilistic, and scenario analyses. This included a value of information (VOI) analysis to quantify the expected value of perfect information (EVPI) surrounding treatment decisions^19,20^.

Our retrospective database analysis and statistical procedures were conducted in SAS 9.4 software (SAS Institute, Raleigh, NC) and Stata 16.0 software (StataCorp, College Station, TX), respectively. The Markov model and Monte Carlo simulations were conducted in Excel and VBA (Microsoft Corporation, Redmond, WA). Graphs were created and our VOI analyses were conducted using the R programming language (R Foundation for Statistical Computing).

### Model

We created a decision analytic Markov model using Microsoft Excel, Visual Basic for Applications (VBA) (Microsoft Corporation, Redmond, WA), and the R programming language (R Foundation for Statistical Computing). Our model evaluates the cost-effectiveness of a hypothetical durable treatment for SCD relative to a current SOC bundle for affected patients in the US. We follow a theoretical cohort over a 100-year horizon with annual cycles to approximate discounted outcomes over a lifetime. As recommended by the Second Panel on Cost-Effectiveness in Health and Medicine, our healthcare sector perspective includes formal medical costs borne by payers and patients^21^. This includes both current and future medical costs related and unrelated to the management of SCD.

Treatment and management of SCD in the United States is complex and varies based on disease severity, patient age, and setting. Despite recent FDA approvals for non-curative treatments voxelotor and crizanlizumab for the management of SCD, the real-world long-term effectiveness, uptake, and patient access to these medications are unknown. For these reasons, we define the SOC as the typical treatment regimen and disease management services received by subgroup in a sample of commercially insured individuals between 2007 and 2017. SOC includes but is not limited to treatment with: antibiotics, vaccinations, pain-relief medications, hydroxyurea, blood transfusions, and stem cell transplants. This data-driven definition for SOC allows for the treatment bundle cost to vary across age, gender, and disease severity levels. Within this group we do not differentiate patients based on specific treatments received, for instance patients treated with hydroxyurea versus those who are not.

For the durable treatment arm, we assume the intervention is a fully effective single administration provided at birth. We define a “fully effective” cure as one that completely suppresses disease-related complications and costs and restores life expectancy and health-related quality of life (HRQoL) to that of a comparable individual unaffected by the disease. Our analysis assumes a cure that is 100% effective across predetermined time ranges (10 years, 20 years, and lifetime). We take an intention-to-treat (ITT) approach in that patients unsuccessful on the durable treatment (i.e., after the treatment has waned) after any period are assumed to move to treatment with the SOC bundle. For this reason, our formal comparison can be stated as two distinct treatment strategies: (1) administration of durable treatment (DT) at birth, with subsequent standard of care management after effectiveness period (henceforth “DT” arm) and (2) standard of care management only (henceforth “SOC” arm). We note that DT is assumed to be 100% effective over a lifetime for all treated patients in our base case analysis. The DT waning assumptions (10 years and 20 years) are tested in scenario analyses.

Our model is informed by various data sources including direct medical costs from a commercial claims retrospective database analysis and other data from the published medical literature. Consistent with recommendations from the second panel on cost-effectiveness in health and medicine, we discount health outcomes and costs at the same rate of 3% per year^21^. Our reporting complies with the Consolidated Health Economic Evaluation Reporting Standards (CHEERS) guidelines where applicable^22^.

### Markov model approach

Markov models are commonly used mathematical tools to simulate recurring events over an extended period of time. Markov models exhibit the “memoryless” property which means transition probabilities depend only on the current state. Informing a Markov model involves providing multiple model inputs. In a health economic evaluation context, these include: health state utility values, costs, and transition probabilities^23^. We outline our methods for obtaining each of these below. A detailed summary of model inputs is available in Table 1.

**Table 1:** Model input parameters and probabilistic sensitivity analysis distributions.

Parameter	Value (95% CI)^a^	Distribution (parameters)	Source
Yearly discount rate	3 (2.4, 3.6)	Not varied in PSA	Assumption
Number of theoretical patients	10,000	Not varied in DSA or PSA	Assumption
Percent female	51 (41.1, 60.9)	Beta (α = 49, β = 47)	Optum 2007–2017
**Initial SCD severities, females**			
Percent mild	50.8		Optum 2007–2017
Percent moderate	22.1	Dirichlet (α_1_ = 227, α_2_ = 99, α_3_ = 121)^b^	Optum 2007–2017
Percent severe	27.1		Optum 2007–2017
**Initial SCD severities, males**			
Percent mild	48.3		Optum 2007–2017
Percent moderate	23.1	Dirichlet (α_1_ = 224, α_2_ = 107, α_3_ = 133)^b^	Optum 2007–2017
Percent severe	28.7		Optum 2007–2017
**QALY weights, females**			
Control QALY weight, ages 1–44	0.89 (0.87, 0.91)	Beta (α = 870.42, β = 107.58)	^24,25^
Control QALY weight, ages 45–54	0.87 (0.85, 0.89)	Beta (α = 983.1, β = 146.9)	^24,25^
Control QALY weight, ages 55–64	0.84 (0.82, 0.86)	Beta (α = 1128.12, β = 214.88)	^24,25^
Control QALY weight, ages 65–74	0.84 (0.82, 0.86)	Beta (α = 1128.12, β = 214.88)	^24,25^
Control QALY weight, ages 75+	0.82 (0.8, 0.84)	Beta (α = 1209.5, β = 265.5)	^24,25^
**QALY weights, males**			
Control QALY weight, ages 1–44	0.89 (0.87, 0.91)	Beta (α = 870.42, β = 107.58)	^24,25^
Control QALY weight, ages 45–54	0.88 (0.86, 0.9)	Beta (α = 928.4, β = 126.6)	^24,25^
Control QALY weight, ages 55–64	0.86 (0.84, 0.88)	Beta (α = 1034.58, β = 168.42)	^24,25^
Control QALY weight, ages 65–74	0.87 (0.85, 0.89)	Beta (α = 983.1, β = 146.9)	^24,25^
Control QALY weight, ages 75+	0.85 (0.83, 0.87)	Beta (α = 1082.9, β = 191.1)	^24,25^
**QALY weights, gender invariant**			
SCD QALY weight, ages 1–18	0.69 (0.57, 0.80)	Normal (μ = 0.688, σ = 0.058)^c^	^25,26^
SCD QALY weight, ages 19+	0.68 (0.67, 0.69)	Normal (μ = 0.682, σ = 0.005)^c^	^25,26^
**Transition probabilities, both genders**			
Ordered logit transition probability regression coefficients, females			
Moderate SCD (α_1_)	1.07 (0.64, 1.51)	Normal (μ = 1.074, σ = 0.223)	Optum 2007–2017
Severe SCD (α_2_)	2.63 (2.19, 3.06)	Normal (μ = 2.625, σ = 0.22)	Optum 2007–2017
Age (α_3_)	− 0.03 (− 0.03, − 0.02)	Normal (μ = − 0.026, σ = 0.004)	Optum 2007–2017
Moderate SCD * age (α_4_)	0.01 (0, 0.02)	Normal (μ = 0.007, σ = 0.006)	Optum 2007–2017
Severe SCD * age (α_5_)	0.02 (0.01, 0.03)	Normal (μ = 0.021, σ = 0.005)	Optum 2007–2017
Cut point 1 (κ_1_)	1.04 (0.76, 1.32)	Normal (μ = 1.04, σ = 0.141)	Optum 2007–2017
Cut point 2 (κ_2_)	1.86 (1.57, 2.15)	Normal (μ = 1.858, σ = 0.147)	Optum 2007–2017
Ordered logit transition probability regression coefficients, males			
Moderate SCD (α_1_)	0.7 (0.23, 1.17)	Normal (μ = 0.701, σ = 0.24)	Optum 2007–2017
Severe SCD (α_2_)	1.98 (1.53, 2.42)	Normal (μ = 1.976, σ = 0.225)	Optum 2007–2017
Age (α_3_)	− 0.03 (− 0.04, − 0.02)	Normal (μ = − 0.03, σ = 0.004)	Optum 2007–2017
Moderate SCD * age (α_4_)	0.01 (0, 0.03)	Normal (μ = 0.012, σ = 0.007)	Optum 2007–2017
Severe SCD * age (α_5_)	0.03 (0.02, 0.05)	Normal (μ = 0.034, σ = 0.007)	Optum 2007–2017
Cut point 1 (κ_1_)	0.79 (0.49, 1.08)	Normal (μ = 0.787, σ = 0.151)	Optum 2007–2017
Cut point 2 (κ_2_)	1.6 (1.3, 1.91)	Normal (μ = 1.605, σ = 0.158)	Optum 2007–2017
**Cost parameters**			
Cost of single administration durable therapy	2.1 M (1.68 M, 2.52 M)	Uniform (a = 1,680,000, b = 2,520,000)	Assumption
**GLM gamma log-link cost regression coefficients, females**			
Intercept (β_0_)	8.05 (7.88, 8.22)	Normal (μ = 8.051, σ = 0.085)	Optum 2007–2017
Mild SCD (β_1_)	1.58 (1.3, 1.85)	Normal (μ = 1.575, σ = 0.139)	Optum 2007–2017
Moderate SCD (β_2_)	1.78 (1.48, 2.09)	Normal (μ = 1.785, σ = 0.154)	Optum 2007–2017
Severe SCD (β_3_)	3.16 (2.88, 3.45)	Normal (μ = 3.165, σ = 0.147)	Optum 2007–2017
Age (β_4_)	0.03 (0.03, 0.04)	Normal (μ = 0.032, σ = 0.002)	Optum 2007–2017
Mild SCD * age (β_5_)	− 0.01 (− 0.02, − 0.01)	Normal (μ = − 0.01, σ = 0.003)	Optum 2007–2017
Moderate SCD * age (β_6_)	− 0.01 (− 0.02, − 0.01)	Normal (μ = − 0.014, σ = 0.003)	Optum 2007–2017
Severe SCD * age (β_7_)	− 0.02 (− 0.03, − 0.02)	Normal (μ = − 0.023, σ = 0.003)	Optum 2007–2017
**GLM gamma log-link cost regression coefficients, males**			
Intercept (β_0_)	8.39 (7.51, 9.28)	Normal (μ = 8.395, σ = 0.452)	Optum 2007–2017
Mild SCD (β_1_)	2.13 (1.16, 3.09)	Normal (μ = 2.126, σ = 0.494)	Optum 2007–2017
Moderate SCD (β_2_)	1.14 (0.21, 2.07)	Normal (μ = 1.14, σ = 0.475)	Optum 2007–2017
Severe SCD (β_3_)	2.36 (1.46, 3.27)	Normal (μ = 2.364, σ = 0.46)	Optum 2007–2017
Age (β_4_)	0.02 (0.01, 0.04)	Normal (μ = 0.024, σ = 0.009)	Optum 2007–2017
Mild SCD * age (β_5_)	− 0.02 (− 0.03, 0)	Normal (μ = − 0.016, σ = 0.009)	Optum 2007–2017
Moderate SCD * age (β_6_)	0.01 (− 0.02, 0.03)	Normal (μ = 0.006, σ = 0.011)	Optum 2007–2017
Severe SCD * age (β_7_)	− 0.01 (− 0.02, 0.01)	Normal (μ = − 0.005, σ = 0.009)	Optum 2007–2017

*PSA* probabilistic sensitivity analysis, *DSA* deterministic sensitivity analysis, *GLM* generalized linear model.

^a^Confidence intervals omitted for variables drawn from multivariate distributions.

^b^Not varied in DSA.

^c^Truncated normal distribution on [0,1].

### Health states

**Figure 1:**
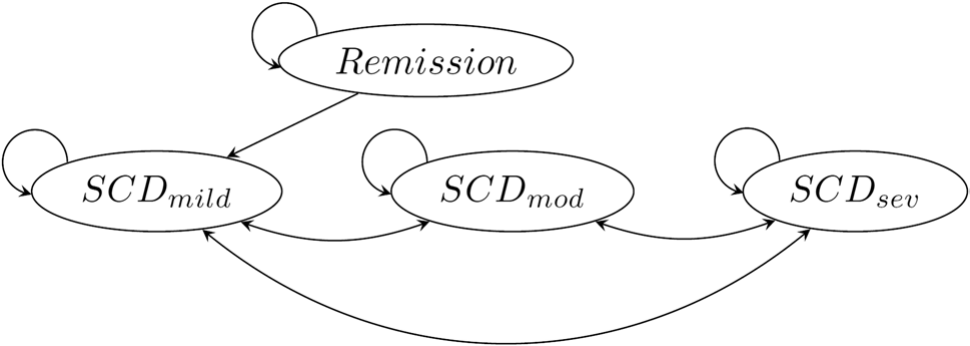
Markov model for lifetime management of SCD. Death (not pictured) can occur from any state. Abbreviations: *SCD* sickle cell disease, *mild* mild disease (zero crises per year), *mod* moderate disease (< 2 crises per year), *sev* severe disease (≥ 2 crises per year).

We employ a Markov model with annual cycles informed by data from a private insurer and the published medical literature to estimate the lifetime direct cost and health-related quality of life under management of SCD (Fig. 1).

Our de novo decision model contains the following health states: (1) healthy, in remission, (2) mild SCD, under SOC treatment, (3) moderate SCD, under SOC treatment, (4) severe SCD, under SOC treatment, and (5) dead. The SCD states are stratified based on the number of crises patients experience on average per year: mild disease (zero crises per year), moderate disease (> 0 but < 2 crises per year), or severe disease (≥ 2 crises per year). For all patients, we assume incidence of sickle cell disease is at birth. We also assume disease-related complications begin to manifest immediately after birth. Given SCD is an inherited genetic condition, these assumptions are consistent with the physiology and subsequent manifestation of the disease^27^. The patient’s initial health state is determined based on treatment arm and subsequent transitions depend on the assumed durability of the single administration therapy, the probability of disease progression, and the probability of death. Initial SCD severity for patients in the SOC arm is based on the observed distributions of mild, moderate, and severe patients observed in our data before age 10. In our base case we assume all patients in the DT arm are treated regardless of gender or disease severity. Death is the only absorbing state in the model.

### Transition probabilities

Patients begin in either the healthy, in remission state (DT arm) or in an affected, on SOC treatment state (SOC arm); see illustration in Fig. 1. Patients in the DT arm continue in remission with a predetermined probability of experiencing relapse. Patients that experience relapse transition to management under SOC with mild disease. These patients may then experience disease progression or death but cannot return to the remission state. In our base case where we assume the durable treatment is effective for a lifetime for all patients who receive it, the probability of relapse is equal to zero.

Patients that begin in the SOC arm stay in an affected state, with zero probability of moving to the healthy state. We use the initial distribution of mild, moderate, and severe disease from our cost data to approximate disease severity in the first year of life. All patients in the model can die after any cycle, with probabilities determined using simulated life tables for black or African American individuals (healthy/remission state) or simulated SCD risk-adjusted life tables (any affected state). Life tables for black or African American individuals are appropriate for our analysis given most cases in the US occur within these groups^7^. We obtain these annual probabilities of death for both arms from a lifetime simulation analysis of patients with and without SCD in the US by Lubeck et al.^25^. The probabilities of disease maintenance or progression are conditional on survival and hence are applied only to patients surviving the cycle.

For patients with SCD, we estimated the probability of moving across disease severities using observed transitions in our data. Using disease severity in years one (severity_1_) and two (severity_2_) we regressed severity_2_ on severity_1_ and age, including interactions. We ran these regressions separately by gender. Our severity classifications are created using an underlying annualized count of vaso-occlusive crises. Consistent with methods the literature, we identified claims with a primary or secondary diagnosis of sickle cell disease with crisis^28^. We then classified patients as having mild disease (zero crises per year), moderate disease (> 0 but < 2 crises per year), or severe disease (≥ 2 crises per year). For this reason, our health states are ordinal and estimation using conditional logistic regression is appropriate^29^. This method for estimating transition probabilities for ordinal health states has been described in detail elsewhere^30^. See Additional File 1 for ordered logistic regression estimates and additional information on transitions.

### Costs

Sickle cell disease is associated with substantial healthcare resource utilization and events for patients managed in the US. Our analysis incorporates regression-estimated direct costs of healthcare associated with managing SCD. For patients in the model treated with SOC, we estimate direct healthcare costs across consecutive age ranges from a retrospective analysis of a large commercial claims database that includes Medicare Advantage patients^31^. For patients in the model who entered with SCD but received DT (i.e., initially assumed cured) and are hence “in remission” we estimate direct costs using data from propensity score matched controls (matched on gender, race, geographic division, year of birth, index year, plan characteristics, and education) unaffected by the disease. Data on these patients are from the same database. See Additional File 1 for further discussion on our cost estimation procedures.

For cured patients we also consider the direct cost of the durable treatment administration. As there are few cell and gene therapies currently available, and none for SCD, there is limited precedent on the potential cost of the product and administration. The most recently approved curative therapy approved by the US Food and Drug Administration for pediatric patients was in spinal muscular atrophy (SMA)^32^. The product, Zolgensma (onasemnogene abeparvovec-xioi), is an adeno-associated (AAV9) virus vector-based gene therapy. It carries a list price of roughly $2.1 million USD as of May 2019^33^. We use this price of $2.1 M as a base case figure for a potential gene therapy in SCD. We vary this price by ± 20% in deterministic (DSA) and probabilistic sensitivity analyses (PSA).

Deceased patients are assumed to incur zero costs. The transition to death also does not incur any costs in the model as to do so would risk double counting. We report all costs in 2018 USD, adjusted by the consumer price index (CPI) or medical component of the CPI when necessary^34^. Future costs related or unrelated to the management of SCD are accounted for through the cohort nature of the model. Every yearly cycle, the patients age an additional year and face modified stage rewards (i.e. costs and utilities) representative of their current state. All future streams are discounted at a rate of 3% per year. See Table 1 for a summary of cost inputs including generalized linear model (GLM) cost regression coefficients by gender and age.

### Health outcomes

Our primary health outcome of interest is average QALYs by treatment arm. We generate QALYs by taking estimated life years lived in each health state and adjusting by a factor within [0,1], also known as a health utility or QALY weight. For our study, we use health utilities (QALY weights) from the published literature. Lubeck et al. provides utilities which vary by age for both patients currently living with SCD and comparable matched controls^25^.

For patients in the “healthy, in remission” health state, we utilize age-specific QALY weights provided in Lubeck et al. which were calculated in Fryback et al. using the EuroQol-5 Dimensions (EQ-5D) instrument to represent a normative US population^24^. For patients in any “affected, under SOC treatment” health state we apply mean health utilities reported in Lubeck et al. for children/adolescents and adults^25^. The authors generated these weights by mapping visual analog pain scale (VAS) scores for patients with SCD to the EQ-5D^26^. We assign deceased patients a QALY weight of zero and discount all future QALYs at a rate of 3% per year. See Table 1 for a summary of utility inputs by treatment arm and age.

### Scenario and sensitivity analyses

In addition to our base case model in which we compare a fully effective durable treatment over a lifetime relative to SOC, we conduct scenario and sensitivity analyses to test the robustness of our results. The primary scenario analyses involve variation in the assumed effectiveness period of the DT. We consider scenarios in which the DT is expected to last for 10 and 20 years for the median patient, after which effects dissipate and disease progression returns to that observed under current SOC. To employ these scenario analyses, we transform the 10- and 20-year probabilities to annual probability of relapse (conditional on staying alive) assuming constant exponential rate. Our equation is:

1 $$\begin{array}{c}{P}_{Annual}=1-{\left(1-{P}_{T years}\right)}^\frac{1}{T}\end{array}$$

Hence for median 10-year duration,

$${P}_{Annual}=1-{\left(1-0.50\right)}^\frac{1}{10}=0.06697$$

and for 20-years

$${P}_{Annual}=1-{\left(1-0.50\right)}^\frac{1}{20}=0.03406$$

In these scenarios the annual probabilities of relapse to mild SCD for patients in the DT arm are 0.06697 and 0.03406, respectively.

In univariate deterministic sensitivity analysis (DSA) we vary each input parameter individually, holding all others constant. We vary parameters within their 95% confidence intervals, or by ± 20% when unavailable. We evaluate impact on the ICER of DT relative to SOC and report results in a tornado diagram. Using the results from univariate DSA, we find the top three nongendered parameters to which the model is most sensitive and vary them by pairs in two-way sensitivity analyses.

In probabilistic sensitivity analysis we fit likely probability distributions to each parameter and conduct 10,000 iterations in a Monte Carlo simulation^35^. Each iteration uses a vector of *K* independent draws, one for each of the *K* input variable’s respective distribution and evaluates them in the model. Following the simulation, we count the number of iterations for which the DT ICER is less than our threshold of $150,000 per QALY and divide by 10,000. We consider this value the percent of the time DT is cost-effective. In addition, we use the results of PSA to compute and graph the cost-effectiveness acceptability curve (CEAC), frontier (CEAF), and the EVPI^19,20,36,37^.

### Ethics approval and consent to participate

Our database analysis component was limited to de-identified data not collected for the purposes of the study. For this reason, the proposal for that study was approved by the University of Southern California’s University Park Institutional Review Board (UPIRB). Our cost-effectiveness study does not utilize human subjects or additional protected health information.

## Results

### Base case and scenario analysis results

**Figure 2:**
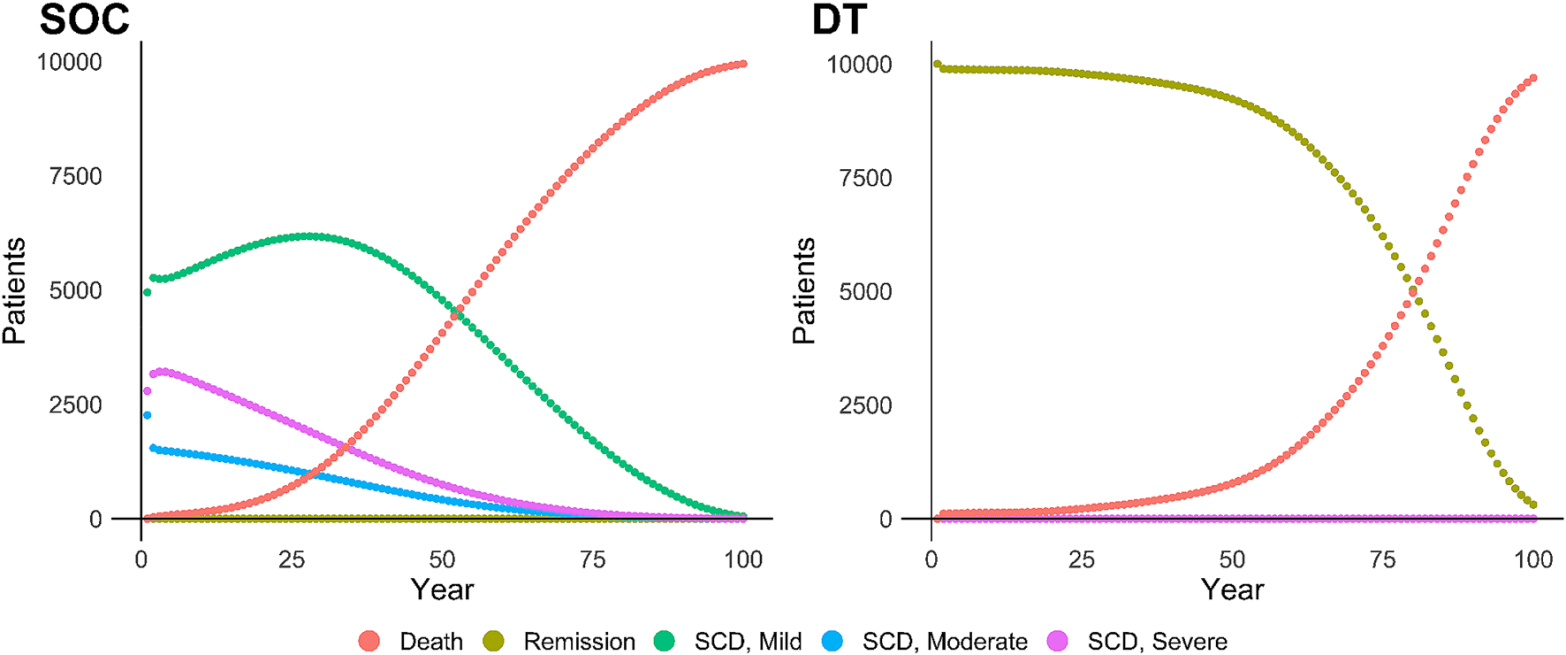
Markov trace for SOC and DT arms (N = 10,000 theoretical patients). *SOC* standard of care, *DT* durable therapy, *SCD* sickle cell disease.

In the base case analysis, we find treatment with DT results in an ICER of $140,877 per QALY and is hence cost-effective at our predetermined WTP threshold of $150K per QALY. DT generated an average of 26.4 discounted QALYs at a discounted cost of $2,372,482 per-patient. SOC resulted in 17.9 discounted QALYs at a discounted cost of $1,175,566 per-patient. Base case results did not differ notably by gender. Females gained an additional 0.6 discounted incremental QALYs relative to males (8.8 vs 8.2) but incurred greater discounted incremental costs of $156,289 over a lifetime relative to males after being treated with DT ($1,279,485 vs $1,123,195). DT ICERs among females and males separately were $146,511 per QALY and $135,574 per QALY, respectively. The health state movements of our theoretical patients are shown in Fig. 2 and detailed base case and scenario analysis results are reported in Table 2.

**Table 2:** Discounted and undiscounted base case and scenario analysis results, per-patient.

	DT				SOC		
	ICER^b^	Cost	QALY	LY	Cost	QALY	LY
**Discounted results**^**a**^							
Base case	$140,877	$2,372,482	26.4	29.9	$1,175,566	17.9	26.2
Females	$146,511	$2,377,583	26.8	30.3	$1,098,098	18.0	26.3
Males	$135,574	$2,367,928	26.1	29.5	$1,244,733	17.9	26.1
DT lasts 20-years^c^	$410,607	$2,761,601	21.8	27.2	$1,175,566	17.9	26.2
Females	$409,161	$2,703,690	22.0	27.4	$1,098,098	18.0	26.3
Males	$411,937	$2,813,307	21.7	27.0	$1,244,733	17.9	26.1
DT lasts 10-years^c^	$740,058	$2,914,175	20.3	26.5	$1,175,566	17.9	26.2
Females	$730,453	$2,836,566	20.4	26.7	$1,098,098	18.0	26.3
Males	$748,848	$2,983,469	20.2	26.4	$1,244,733	17.9	26.1
**Undiscounted results**							
Base case	$15,332	$3,210,182	66.2	75.8	$2,770,348	37.6	54.9
Females	$24,825	$3,364,554	68.5	78.9	$2,607,231	38.0	55.6
Males	$5,778	$3,072,350	64.2	73.0	$2,915,988	37.2	54.3
DT lasts 20-years^c^	$162,116	$4,154,316	46.1	59.6	$2,770,348	37.6	54.9
Females	$163,939	$4,054,067	46.8	60.7	$2,607,231	38.0	55.6
Males	$160,380	$4,243,825	45.4	58.7	$2,915,988	37.2	54.3
DT lasts 10-years^c^	$419,576	$4,411,570	41.5	56.3	$2,770,348	37.6	54.9
Females	$411,772	$4,257,316	42.0	57.1	$2,607,231	38.0	55.6
Males	$426,872	$4,549,296	41.0	55.6	$2,915,988	37.2	54.3

*DT* durable therapy, *SOC* standard of care, *ICER* incremental cost-effectiveness ratio, *QALY* quality-adjusted life year, *LY* life year.

^a^All outcomes discounted at 3% per year.

^b^ICER units are 2018 US dollars per QALY.

^c^Expected median duration.

Our scenario analyses involved relaxing the assumption that DT will last a lifetime for all patients treated. In our median 20-year duration scenario the health outcomes of DT dropped to an average of 21.8 discounted QALYs gained with an increase in discounted cost to $2,761,601 due to unsuccessful patients moving to treatment with SOC. The ICER in this scenario was $410,607 per QALY. A scenario in which DT lasts a median of 10 years further decreases QALYs, increases costs, and results in an ICER of $740,058 per QALY. We do not consider DT to be cost-effective in either of these scenarios.

### Deterministic sensitivity analysis

In DSA we first varied parameters one at a time within their 95% confidence intervals (± 20% when unavailable) to study impact on the base case ICER ($140,877/QALY). We find the model to be most sensitive to variations in two of the coefficients in the cost regression of treating males, DT treatment cost, the discount rate, and the QALY weight for children and adolescents with SCD. Univariately, the model is most sensitive to the regression coefficients $${\widehat{\beta }}_{0}$$ and $${\widehat{\beta }}_{1}$$ in the GLM regressions of cost on observable characteristics for males. Decreasing (increasing) $${\widehat{\beta }}_{0}$$ within its 95% CI results in an ICER for DT of $176,589 ($54,207) per QALY. Similarly, decreasing (increasing) $${\widehat{\beta }}_{1}$$ within its 95% CI results in an ICER for DT of $169,491 ($65,582) per QALY. These results are intuitive—all else equal, increasing $${\widehat{\beta }}_{0}$$ increases the predicted cost gap between male control patients and male patients with SCD, of any severity. Similarly, increasing $${\widehat{\beta }}_{1}$$ increases the cost gap between male control patients and specifically male patients with mild SCD. In both scenarios the cost-effectiveness of a durable cure relative to standard of care increases.

**Figure 3:**
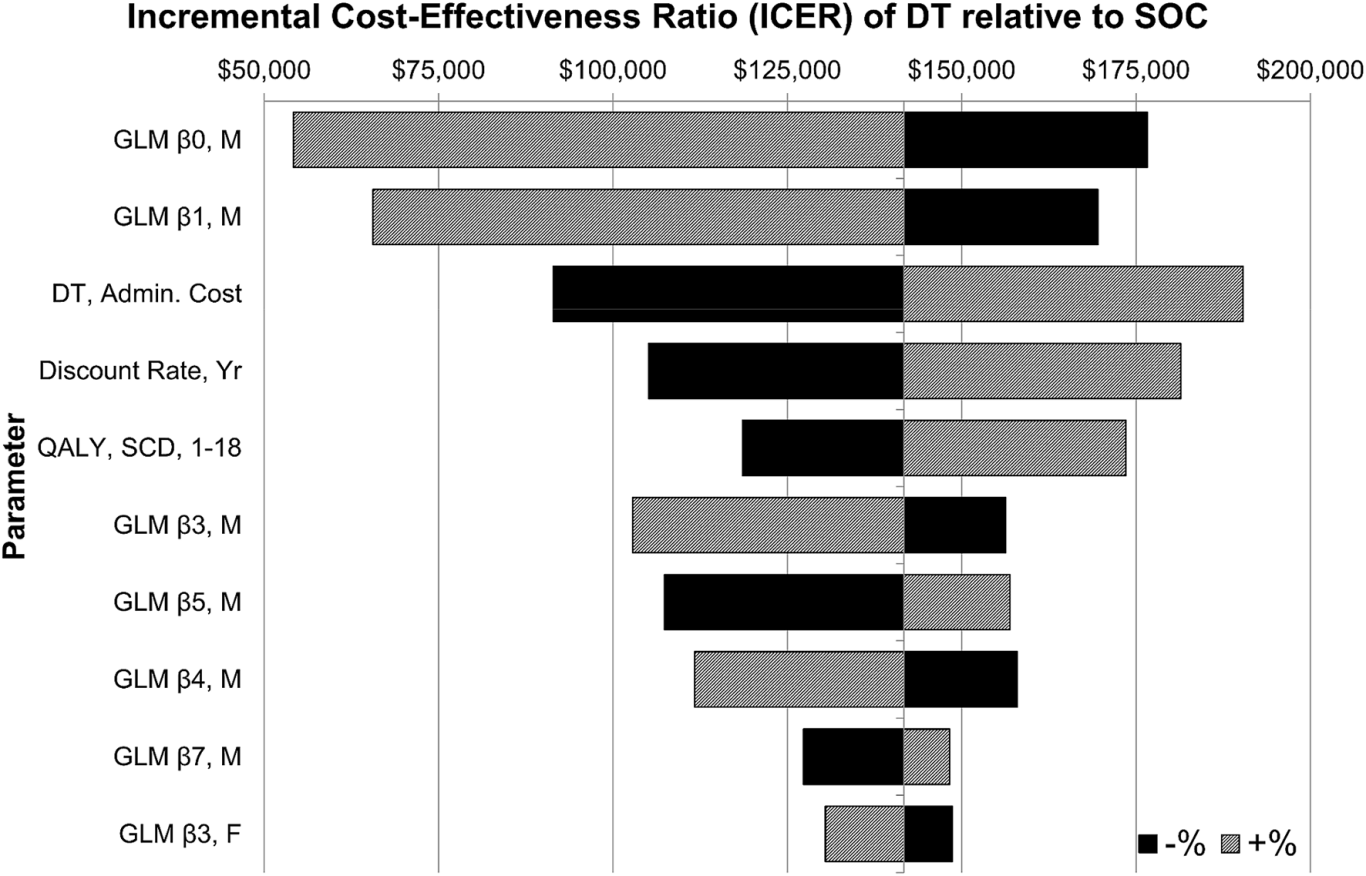
Tornado diagram of deterministic sensitivity analysis results (top ten). Parameters were varied univariately within their 95% CIs, or ± 20% when unavailable. *CI* confidence interval, *SOC* standard of care, *DT* durable therapy, *SCD* sickle cell disease, *Yr* year; *QALY*, quality-adjusted life year, *GLM* generalized linear model, *M* males, *F* females, *O. Logit* ordered logistic regression, *Cont.* control patients, *Prop.* proportion.

Decreasing (increasing) the DT treatment cost (base value $2.1 M USD) by 20 percent resulted in an ICER for DT of $91,443/QALY ($190,311/QALY). Changes in the DT price result in pronounced impact on the model ICER due to the nature of upfront payment; in the model patients are treated immediately and hence the cost is not subject to discounting over future periods. Similarly, decreasing (increasing) the discount rate (base value 3%) by 20% resulted in an ICER for DT of $105,118/QALY ($181,407/QALY). This result suggests that a health system that is less interested about future costs and effects is less likely to find DT to be cost-effective. This is because single administration costs are upfront while benefits are disproportionately longer term. Lastly, setting the SCD QALY weight for children/adolescents at its lower and upper 95% CI bounds resulted in ICERs for DT of $118,559 per QALY and $173,546 per QALY, respectively. All else equal, patients with SCD who have their condition managed well may be less cost-effective to treat than more severe cases. This is especially true for children and adolescents as health gains during the first two decades of life are discounted the least in the model. A tornado diagram of the top ten parameters in DSA is shown in Fig. 3. See Supplementary Information: Figure S9 for a complete tornado diagram and Table S1 for a corresponding table of DSA results including variation ranges.

**Figure 4:**
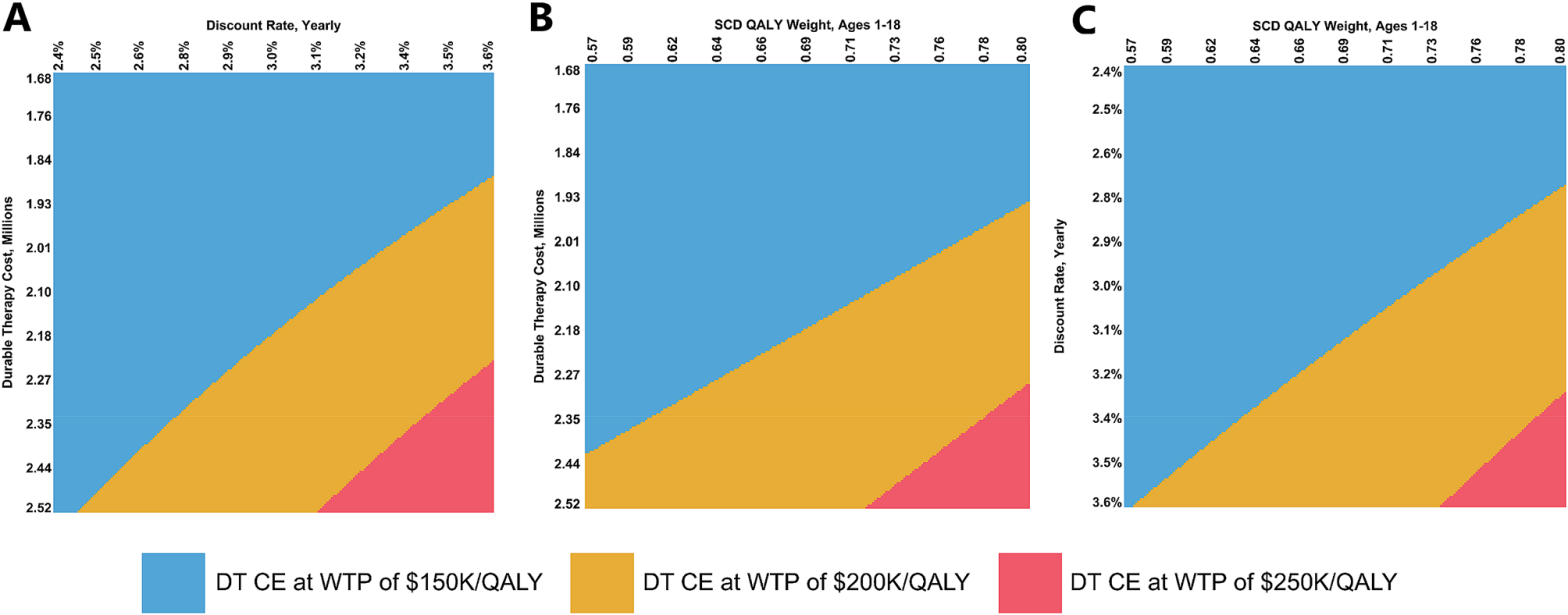
Two-way deterministic sensitivity analyses of select model parameters. (**A**) DT upfront cost versus yearly discount rate. (**B**) DT upfront cost versus pediatric QALY weight. (**C**) Yearly discount rate versus pediatric QALY weight. Parameters were varied simultaneously within their 95% CIs, or ± 20% when unavailable. *DT*, durable therapy; *QALY*, quality-adjusted life year; *CI*, confidence interval; *CE*, cost-effective; *WTP*, willingness to pay; *K*, thousand.

Using the results from univariate DSA, we identified the top three non-gender specific parameters to which the model ICER was most sensitive. These include the upfront cost of DT, the discount rate, and the QALY weight for children and adolescents with SCD. We then varied these in three different two-way deterministic sensitivity analyses: DT price versus discount rate, DT price versus pediatric/adolescent QALY weight, and discount rate versus pediatric/adolescent QALY weight. Findings were similar in all three comparisons. Slight simultaneous increases in these parameters often resulted in an ICER for DT between $150,000 and $200,000/QALY. In all three two-way DSAs, DT was cost-effective at WTP of $250K or higher per QALY regardless of parameter values. See Fig. 4 for detailed graphs of two-way DSA.

### Probabilistic sensitivity analysis and value of information

**Figure 5:**
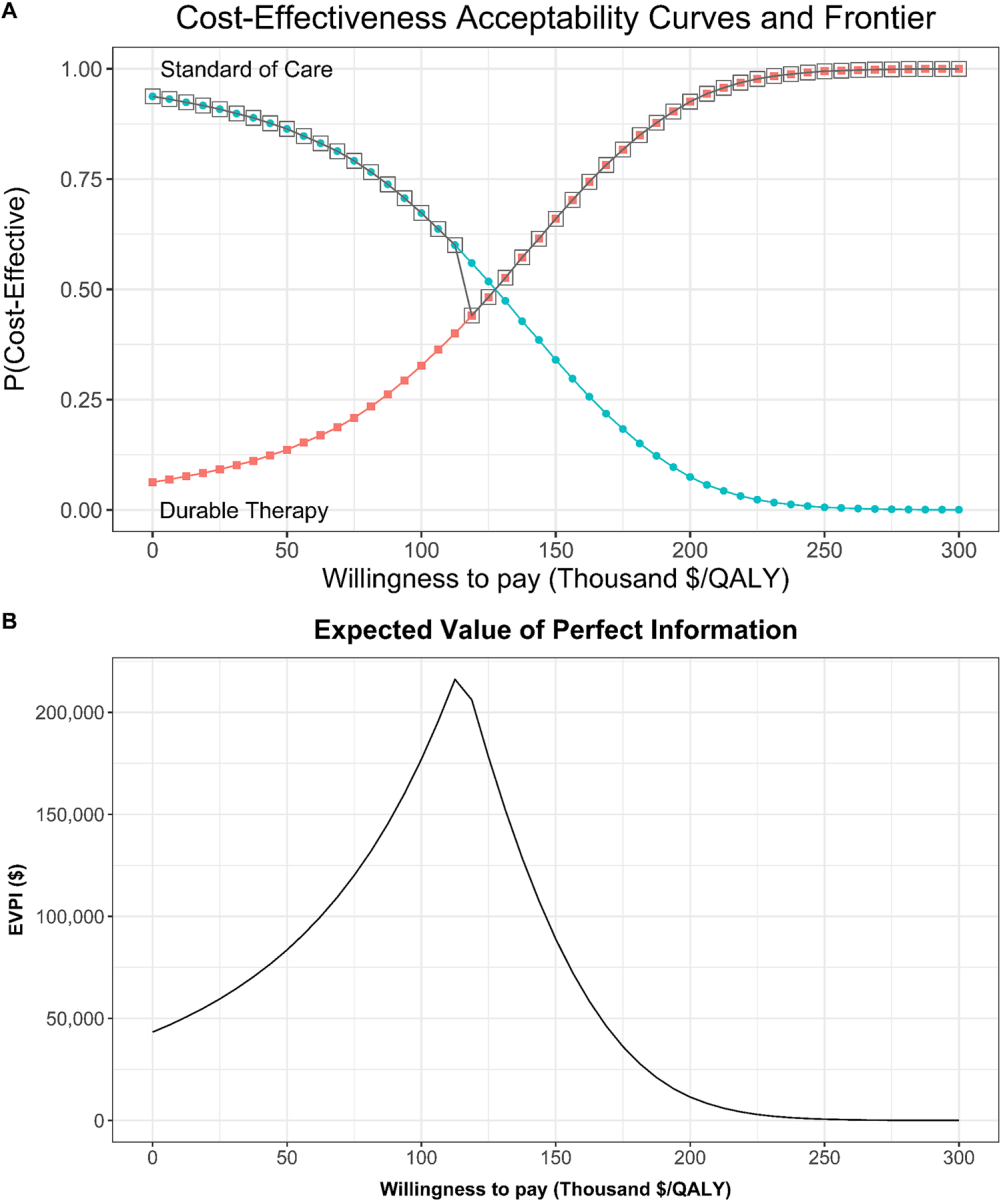
Probabilistic sensitivity analysis results. (**A**) Cost-effectiveness acceptability curves (CEAC) and frontier (CEAF). (**B**) Expected value of perfect information (EVPI).

Our primary outcome of interest in PSA was the ICER for DT relative to SOC. In 10,000 iterations, DT was cost-effective relative to SOC 13.6%, 32.7%, 66.0%, and 92.6% of the time at WTP values of $50K, $100K, $150K, and $200K/QALY, respectively (Fig. 5). Across genders, DT was much more likely to be cost-effective at $100K WTP per QALY for males (42.5%) than for females (11.1%). See Additional File 1: Figures S10 and S11 for gender-specific results.

In value of information analysis the expected value of perfect information (EVPI) for the average patient is highest at the WTP associated with the highest decision uncertainty. In our model, this occurs at a WTP per QALY of $115K where both arms have an equal probability of being cost-effective. At this WTP, the EVPI is $224.1K per-patient suggesting this is the most a healthcare system should be willing to pay to eliminate model and parameter uncertainty. At our predetermined WTP threshold of $150K/QALY, the EVPI is $88.9K per patient (Fig. 5).

## Discussion and limitations

Based on currently available information on the economic and health-related quality of life burdens, our findings suggest that a durable treatment or cure for sickle cell disease would be cost-effective to the US healthcare sector. Assuming a $2.1 million USD all-inclusive price for treatment, the average patient with SCD cured at birth stands to gain 8.5 QALYs and 3.7 LYs over a lifetime, at an incremental cost of $1,196,917 (all values discounted). As DT leads to both an increase in life expectancy as well as a marked improvement in utility over the patient’s lifetime, the incremental QALY and incremental LY values are positive, with the QALY gain exceeding the LY gain. This results in a base case ICER from our model of $140,877 per QALY for an initial distribution of 47% females (51% mild SCD, 22% moderate SCD, 27% severe SCD), and 53% males (48% mild SCD, 23% moderate SCD, 29% severe SCD). These initial gender and severity distributions were approximated using our data.

We do not find substantial differences in base case cost-effectiveness results across subgroups. The ICERs for curing females and males in our base case are $146,511 per QALY and $135,574 per QALY, respectively. However, in scenario analyses, we find that cost-effectiveness is highly contingent on the single administration therapy being fully effective for the lifetime of the patient. For a scenario in which patients have a 50% chance of relapse (conditional on survival) by 20 years, the overall ICER increases to $410,607 per QALY. If we change this value to 10 years, the ICER further increases to $740,058 per QALY.

Our sensitivity analysis results suggest the likelihood DT is not cost-effective decreases dramatically beyond willingness to pay of $250K/QALY. In univariate DSA, the largest ICER we obtained was $190,311 per QALY from increasing the durable treatment price to $2.52 million USD. In two-way DSA we find that slight simultaneous increases our baseline discount rate and upfront cost of DT would result in an ICER for DT of over $150K/QALY; however, no comparisons resulted in an ICER above $250K/QALY. The same result holds true for pairwise comparisons of upfront cost of DT versus the pediatric/adolescent SCD QALY weight and discount rate versus the pediatric/adolescent SCD QALY weight. In PSA, DT was cost-effective 32.7%, 66.0%, and 92.6% of the time at WTP values of $100K, $150K, and $200K/QALY, respectively. Through VOI analysis we find that a health system would be willing to pay up to approximately $224.1K per-patient to eliminate all uncertainty regarding durable treatment decisions.

Our cost-effectiveness study is not without limitations. We assume treatment for the durable therapy arm is provided at birth, which may not align with true eligible population. Given the treatment is hypothetical, it is unclear who will qualify for the treatment and subsequently receive it. In addition, we assume the cure is single administration, and patients cannot receive it twice. A recently FDA approved gene therapy in SMA, onasemnogene abeparvovec-xioi, is a one-time administration through intravenous therapy available to children younger than two years^38^. We assume the treatment is 100% effective during the durability period. This period is lifetime in our base case, but we vary it between a median of 10 and 20 years in our scenario analyses.

The data on costs in our study are appropriate for our purposes but carry limitations. The direct costs are estimated from a retrospective database analysis of the commercially insured, and do not vary based on time from death. They are based on estimated allowed amounts, or ultimately what the insurer agrees to pay for services. A societal perspective analysis would include other direct costs not borne by the healthcare sector. Our assumed cost for a single administration durable therapy is based on the most recently available comparable product. We use Zolgensma (onasemnogene abeparvovec-xioi), a gene therapy approved for the treatment of pediatric SMA, which carries a list price of $2.1 million USD. This price may not account for discounts, rebates, or other costs associated with treatment administration.

In addition, the use of Zolgensma’s price may complicate our analysis if it was determined by market forces and the presumptive value it brings to patients with SMA. To address this, we note that an independent review by the Institute for Clinical and Economic Review (ICER) found that Zolgensma’s incremental cost-effectiveness ratio relative to best supportive care (BSC) for treating SMA type I would be roughly $243,000 per QALY at a hypothetical placeholder price of $2.0 million USD^39^. If we assume the current market price was informed by this analysis, a $2.1 million USD price tag (+ $100,000 to their placeholder price), would imply the price was justified based on an ICER of roughly $252,000 per QALY. This is because Zolgensma generated 11.77 QALYs at an increased cost of $2.87 M USD over BSC. If price increases $100K, the new ICER for Zolgensma is $2.97 M/11.77 QALYs or approximately $252K/QALY. For our purposes, an ICER of $252,000 per QALY would correspond with a single administration total cost of $3.04 million USD for a durable SCD cure; however, we would not consider the therapy to be cost-effective at this implied price point.

We do not incorporate indirect costs of managing SCD. These may include costs such as uncompensated caregiver burden, lost income to presenteeism or absenteeism, and other indirect costs borne by the healthcare or other sectors. However, indirect costs are likely to be larger under SOC than in the DT arm due to the well-documented humanistic burden associated with managing SCD. This means our ICER likely underestimates the true benefit of a durable therapy; we would expect significant productivity benefits from the cure of a chronic disease. In addition, we would also expect greater lifetime income from DT due to longer lifespan and additional working years.

Our base case analysis uses a 3% discount rate on both costs and health outcomes, a number based on the estimated real consumption rate of interest. This discounting procedure is recommended by the Second Panel on Cost Effectiveness in Health and Medicine^21^. However, appropriate methods for discounting in health economic evaluation are still a subject of debate^40,41^. As confirmed in our case using sensitivity analysis results, a high discount rate has a disproportionate impact on the incremental cost-effectiveness ratio of therapies for which (i) the majority of costs are upfront and (ii) the majority of health benefits are downstream. Despite a likely large upfront cost, a durable therapy for SCD would provide significant health gains for patients over the course of an extended lifespan. We acknowledge the impact discount rate has on study results and for this reason also report undiscounted costs, life years, quality-adjusted life years, and ICERs for additional interpretation.

Lastly, gene therapy acceptability among patients with SCD in the US is unclear^42^. Any treatment that becomes available to the US market will have satisfied FDA standards for safety. Despite this, elective uptake at the patient level is uncertain. The black and African American community has been shown to exhibit high levels of medical mistrust, often attributed to a vast history of marginalization and medical exploitation in the United States^43^. In SCD specifically, there is evidence that a common barrier to allogenic HSCT clinical trial participation is mistrust of healthcare providers^44^. Cell or gene therapy uptake for appropriate patients may require informational campaigns or other strategies to directly address mistrust in the healthcare system^45^.

## Conclusions

We used a decision analytic model to explore the cost-effectiveness of a hypothetical durable cure for sickle cell disease in the US. Our base case model suggests treatment is likely cost-effective, contingent on durability of the cure. In probabilistic sensitivity analysis durable therapy is cost effective 32.7%, 66.0%, and 92.6% of the time at WTP values of $100,000, $150,000, and $200,000 per QALY, respectively. We find durable treatment would be cost-effective at a minimum willingness to pay (WTP) of $150,000 per QALY at single administration costs of $713K, $1.09 M, and $2.18 M, for cure durations of median 10 years, median 20 years, and lifetime, respectively. We acknowledge our findings may not be applicable to all patients affected by SCD in the US. There is substantial heterogeneity in SCD manifestation and subsequent treatment. Despite this, understanding potential uptake and impacts prior to treatment availability is difficult. Like HSCT, gene therapy treatment may be reserved for the most severe patients who have failed numerous lines of previous therapies. In addition, gene therapy acceptability may be low among the SCD community. We build our model based on available data with the potential to provide refined estimates for specific groups as a cure becomes likely. We hope additional data on the economic and clinical burdens of managing sickle cell disease in the US are forthcoming.

## Supplementary Information

Supplementary Information.

## Data Availability

The data on commercially insured patients (Optum’s de-identified Clinformatics Data Mart Database) are not publicly available. We used these data under license; they are available from Optum for a fee. All other data generated or analyzed during this study are cited or included in this published article and its supplementary information files.

## Abbreviations

CE: Cost-effective
CEAC: Cost-effectiveness acceptability curve
CEAF: Cost-effectiveness acceptability frontier
CHEERS: Consolidated Health Economic Evaluation Reporting Standards
CI: Confidence interval
CONT: Continued
CPI: Consumer price index
DSA: Deterministic sensitivity analysis
DT: Durable therapy
EQ-5D: EuroQol 5-dimensions
EVPI: Expected value of perfect information
FDA: Food and Drug Administration
GDP: Gross domestic product
GLM: Generalized linear model
HSCT: Hematopoietic stem cell transplant
ICER: Incremental cost-effectiveness ratio
ICER: Institute for Clinical and Economic Review
ITT: Intention-to-treat
LR: Likelihood ratio
LY: Life year
MIL: Million
NB: Net benefit
PSA: Probabilistic sensitivity analysis
QALY: Quality-adjusted life year
SCA: Sickle cell anemia
SCD: Sickle cell disease
SE: Standard error
SMA: Spinal muscular atrophy
SOC: Standard of care
UPIRB: University Park Institutional Review Board
US: United States of America
USD: US dollar
VAS: Visual analog scale
VBA: Visual Basic for Applications
VOC: Vaso-occlusive crisis
VOI: Value of information
WTP: Willingness-to-pay

## References

[1] Rees DC, Williams TN, Gladwin MT (2010). Sickle-cell disease. Lancet.

[2] Sundd P, Gladwin MT, Novelli EM (2019). Pathophysiology of sickle cell disease. Annu. Rev. Pathol..

[3] Vekilov PG (2007). Sickle-cell haemoglobin polymerization: Is it the primary pathogenic event of sickle-cell anaemia?. Br. J. Haematol..

[4] Platt OS (1994). Mortality in sickle cell disease. Life expectancy and risk factors for early death. N. Engl. J. Med..

[5] National Heart, Lung & and Blood Institute (2014). Evidence-Based Management of Sickle Cell Disease: Expert Panel report, 2014.

[6] Platt OS (1991). Pain in sickle cell disease. Rates and risk factors. N. Engl. J. Med..

[7] Hassell KL (2010). Population estimates of sickle cell disease in the U.S. Am. J. Prev. Med..

[8] Meier ER (2018). Treatment options for sickle cell disease. Pediatr. Clin. North Am..

[9] Nevitt SJ, Jones AP, Howard J (2017). Hydroxyurea (hydroxycarbamide) for sickle cell disease. Cochrane Database Syst. Rev..

[10] Lanzkron S, Haywood C, Segal JB, Dover GJ (2006). Hospitalization rates and costs of care of patients with sickle-cell anemia in the state of Maryland in the era of hydroxyurea. Am. J. Hematol..

[11] Kapoor S, Little JA, Pecker LH (2018). Advances in the treatment of sickle cell disease. Mayo Clin. Proc..

[12] Kassim AA, Sharma D (2017). Hematopoietic stem cell transplantation for sickle cell disease: The changing landscape. Hematol. Oncol. Stem Cell Ther..

[13] Vichinsky E (2019). A phase 3 randomized trial of voxelotor in sickle cell disease. N. Engl. J. Med..

[14] Ataga KI (2017). Crizanlizumab for the prevention of pain crises in sickle cell disease. N. Engl. J. Med..

[15] U.S. Food and Drug Administration. *FDA approves voxelotor for sickle cell disease*, https://www.fda.gov/drugs/resources-information-approved-drugs/fda-approves-voxelotor-sickle-cell-disease (2019).

[16] U.S. Food and Drug Administration. *FDA approves first targeted therapy to treat patients with painful complication of sickle cell disease*, https://www.fda.gov/news-events/press-announcements/fda-approves-first-targeted-therapy-treat-patients-painful-complication-sickle-cell-disease (2019).

[17] Rubin R (2019). Gene therapy for sickle cell disease shows promise. JAMA.

[18] Neumann PJ, Cohen JT, Weinstein MC (2014). Updating cost-effectiveness–the curious resilience of the $50,000-per-QALY threshold. N. Engl. J. Med..

[19] Fenwick E (2020). Value of information analysis for research decisions-an introduction: Report 1 of the ISPOR value of information analysis emerging good practices task force. Value Health.

[20] Rothery C (2020). Value of information analytical methods: Report 2 of the ISPOR value of information analysis emerging good practices task force. Value Health.

[21] Neumann PJ, Sanders GD, Russell LB, Siegel JE, Ganiats TG (2017). Cost Effectiveness in Health and Medicine.

[22] Husereau D (2013). Consolidated Health Economic Evaluation Reporting Standards (CHEERS)–explanation and elaboration: A report of the ISPOR health economic evaluation publication guidelines good reporting practices task force. Value Health.

[23] Denton BT (2013). Handbook of Healthcare Operations Management: Methods and Applications.

[24] Fryback DG (2007). US norms for six generic health-related quality-of-life indexes from the National Health Measurement study. Med. Care.

[25] Lubeck D (2019). Estimated life expectancy and income of patients with sickle cell disease compared with those without sickle cell disease. JAMA Netw. Open.

[26] Anie KA (2012). Patient self-assessment of hospital pain, mood and health-related quality of life in adults with sickle cell disease. BMJ Open.

[27] Hall JB, Kress J, Schmidt GA (2015). Principles of Critical Care.

[28] Shah N, Bhor M, Xie L, Paulose J, Yuce H (2019). Sickle cell disease complications: Prevalence and resource utilization. PLoS ONE.

[29] Cameron AC, Trivedi PK (2010). Microeconometrics using Stata.

[30] Jung, J. Estimating Markov transition probabilities between health states in the HRS dataset. (2006).

[31] Optum.com. *Optum Research Data Assets.* https://www.optum.com/content/dam/optum/resources/productSheets/5302_Data_Assets_Chart_Sheet_ISPOR.pdf.

[32] U.S. Food and Drug Administration. *FDA approves innovative gene therapy to treat pediatric patients with spinal muscular atrophy, a rare disease and leading genetic cause of infant mortality*, https://www.fda.gov/news-events/press-announcements/fda-approves-innovative-gene-therapy-treat-pediatric-patients-spinal-muscular-atrophy-rare-disease (2019).

[33] Rosenmayr-Templeton L (2019). Industry update for May 2019. Ther. Deliv..

[34] U.S. Bureau of Labor Statistics. *Medical care in U.S. city average*, https://data.bls.gov/timeseries/CUUR0000SAM?output_view=pct_12mths (2019).

[35] Hatswell AJ, Bullement A, Briggs A, Paulden M, Stevenson MD (2018). Probabilistic sensitivity analysis in cost-effectiveness models: Determining model convergence in cohort models. Pharmacoeconomics.

[36] van Hout BA, Al MJ, Gordon GS, Rutten FF (1994). Costs, effects and C/E-ratios alongside a clinical trial. Health Econ..

[37] Fenwick E, Claxton K, Sculpher M (2001). Representing uncertainty: The role of cost-effectiveness acceptability curves. Health Econ..

[38] Zolgensma-one-time gene therapy for spinal muscular atrophy. *Med. Lett. Drugs Ther.* **61**, 113–114 (2019). 31381549

[39] Pearson SD, Thokala P, Stevenson M, Rind D (2019). The effectiveness and value of treatments for spinal muscular atrophy: A summary from the institute for clinical and economic review’s New England Comparative Effectiveness Public Advisory Council. J. Manag. Care Specialty Pharm..

[40] Attema AE, Brouwer WBF, Claxton K (2018). Discounting in economic evaluations. Pharmacoeconomics.

[41] Claxton K, Paulden M, Gravelle H, Brouwer W, Culyer AJ (2011). Discounting and decision making in the economic evaluation of health-care technologies. Health Econ..

[42] Strong H (2017). Patient perspectives on gene transfer therapy for sickle cell disease. Adv. Ther..

[43] Alsan M, Wanamaker M (2017). Tuskegee and the health of black men*. Q. J. Econ..

[44] Omondi NA (2013). Barriers to hematopoietic cell transplantation clinical trial participation of African American and black youth with sickle cell disease and their parents. J. Pediatr. Hematol. Oncol..

[45] Stevens EM, Patterson CA, Li YB, Smith-Whitley K, Barakat LP (2016). Mistrust of pediatric sickle cell disease clinical trials research. Am. J. Prev. Med..

