# Supplementary material for: Cost-effectiveness of a hypothetical cell or gene therapy cure for sickle cell disease
Source: Sci Rep. 2021 May 25;11:10838. doi: 10.1038/s41598-021-90405-1 (PMC8149675; doi:10.1038/s41598-021-90405-1)
Supplement: Supplementary file 1 — Supplementary Information. [file 41598_2021_90405_MOESM1_ESM.docx]

**Supplementary Information for Cost-Effectiveness of a Hypothetical Cell or Gene Therapy Cure for Sickle Cell Disease**

**Authors and affiliations**

Jonathan Salcedo, PhD^1,2^, Jenniffer Bulovic, MD MPH^2^, Colin M. Young, PhD^2^

^1^ Department of Pharmaceutical and Health Economics, School of Pharmacy, University of Southern California, Los Angeles, CA, USA

^2^ Center for Biomedical Innovation, Massachusetts Institute of Technology, Cambridge, MA, USA

| **Supplementary Table 1** Univariate deterministic sensitivity analysis results on ICER, ordered by base case model sensitivity | | | | | |
| --- | --- | --- | --- | --- | --- |
| Parameter | -% | +% | Range | Base | Variation |
| GLM β0, M | $176,589 | $54,207 | $122,381 | 8.39 | 10.56 |
| GLM β1, M | $169,491 | $65,582 | $103,909 | 2.13 | 45.52 |
| DT, Admin. Cost | $91,443 | $190,311 | $98,868 | 2100000.00 | 20.00 |
| Discount Rate, Yr | $105,118 | $181,407 | $76,289 | 0.03 | 20.00 |
| QALY, SCD, 1-18 | $118,559 | $173,546 | $54,988 | 0.69 | 16.64 |
| GLM β3, M | $156,301 | $102,850 | $53,451 | 2.36 | 38.17 |
| GLM β5, M | $107,394 | $156,913 | $49,518 | -0.02 | 115.96 |
| GLM β4, M | $157,938 | $111,699 | $46,240 | 0.02 | 71.94 |
| GLM β7, M | $127,321 | $148,280 | $20,959 | -0.01 | 337.68 |
| GLM β3, F | $148,670 | $130,489 | $18,181 | 3.16 | 9.08 |
| GLM β0, F | $147,896 | $132,580 | $15,315 | 8.05 | 2.08 |
| GLM β1, F | $146,900 | $132,974 | $13,926 | 1.58 | 17.25 |
| O. Logit κ2, F | $134,389 | $146,335 | $11,946 | 1.86 | 15.52 |
| GLM β2, M | $144,086 | $132,739 | $11,347 | 1.14 | 81.61 |
| O. Logit α2, F | $144,920 | $135,702 | $9,218 | 2.63 | 16.44 |
| QALY, M, Cont., 1-44 | $145,361 | $136,662 | $8,699 | 0.89 | 2.29 |
| GLM β5, F | $136,116 | $144,633 | $8,518 | -0.01 | 50.38 |
| O. Logit κ2, M | $136,338 | $144,504 | $8,166 | 1.60 | 19.28 |
| QALY, F, Cont., 1-44 | $144,902 | $137,070 | $7,832 | 0.89 | 2.29 |
| GLM β7, F | $136,636 | $144,344 | $7,708 | -0.02 | 29.32 |
| O. Logit α3, F | $137,182 | $143,779 | $6,597 | -0.03 | 26.66 |
| GLM β6, M | $142,842 | $136,344 | $6,499 | 0.01 | 343.60 |
| GLM β4, F | $143,522 | $138,015 | $5,507 | 0.03 | 10.85 |
| O. Logit α3, M | $137,732 | $142,873 | $5,141 | -0.03 | 28.05 |
| O. Logit α2, M | $142,660 | $138,343 | $4,317 | 1.98 | 22.35 |
| O. Logit α1, F | $142,670 | $138,586 | $4,084 | 1.07 | 40.79 |
| O. Logit α5, F | $142,666 | $138,646 | $4,020 | 0.02 | 52.08 |
| QALY, SCD, 19+ | $138,979 | $142,828 | $3,850 | 0.68 | 1.39 |
| O. Logit α5, M | $142,183 | $138,969 | $3,215 | 0.03 | 37.55 |
| O. Logit κ1, F | $139,200 | $142,376 | $3,176 | 1.04 | 26.64 |
| GLM β2, F | $142,038 | $139,307 | $2,731 | 1.78 | 16.92 |
| Initial Prop. Female | $139,832 | $141,912 | $2,081 | 0.47 | 20.12 |
| O. Logit α4, F | $141,644 | $139,907 | $1,737 | 0.01 | 156.54 |
| O. Logit κ1, M | $141,738 | $140,051 | $1,687 | 0.79 | 37.70 |
| O. Logit α1, M | $141,451 | $139,991 | $1,460 | 0.70 | 67.13 |
| GLM β6, F | $140,188 | $141,428 | $1,241 | -0.01 | 45.37 |
| O. Logit α4, M | $141,320 | $140,178 | $1,142 | 0.01 | 113.13 |
| QALY, M, Cont., 45-54 | $141,263 | $140,493 | $770 | 0.88 | 2.30 |
| QALY, F, Cont., 45-54 | $141,235 | $140,521 | $714 | 0.87 | 2.32 |
| QALY, M, Cont., 55-64 | $141,137 | $140,618 | $519 | 0.86 | 2.34 |
| QALY, F, Cont., 55-64 | $141,126 | $140,629 | $497 | 0.84 | 2.39 |
| QALY, F, Cont., 65-74 | $141,040 | $140,714 | $326 | 0.84 | 2.39 |
| QALY, M, Cont., 65-74 | $141,034 | $140,720 | $314 | 0.87 | 2.32 |
| QALY, F, Cont., 75+ | $141,011 | $140,743 | $268 | 0.82 | 2.44 |
| QALY, M, Cont., 75+ | $140,980 | $140,774 | $206 | 0.85 | 2.37 |

Base case ICER was $140,877/QALY. ICER units are dollars per QALY. Parameters were varied univariately within their 95% CIs, or ± 20% when unavailable. Abbreviations: ICER, incremental cost-effectiveness ratio; CI, confidence interval; SOC, standard of care; DT, durable therapy; SCD, sickle cell disease; Yr, year; QALY, quality-adjusted life year; GLM, generalized linear model; M, males; F, females; O. Logit, ordered logistic regression; Cont., control patients; Prop., proportion.

**Supplementary Figure 1** Ordered logit predicted probabilities of transitioning to mild SCD by gender, age, and state

Abbreviations: SCD, sickle cell disease.

**Supplementary Figure 2** Ordered logit predicted probabilities of transitioning to moderate SCD by gender, age, and state

Abbreviations: SCD, sickle cell disease.

**Supplementary Figure 3** Ordered logit predicted probabilities of transitioning to severe SCD by gender, age, and state

Abbreviations: SCD, sickle cell disease.

**Supplementary Figure 4** Markov trace for SOC and DT arms, females

**
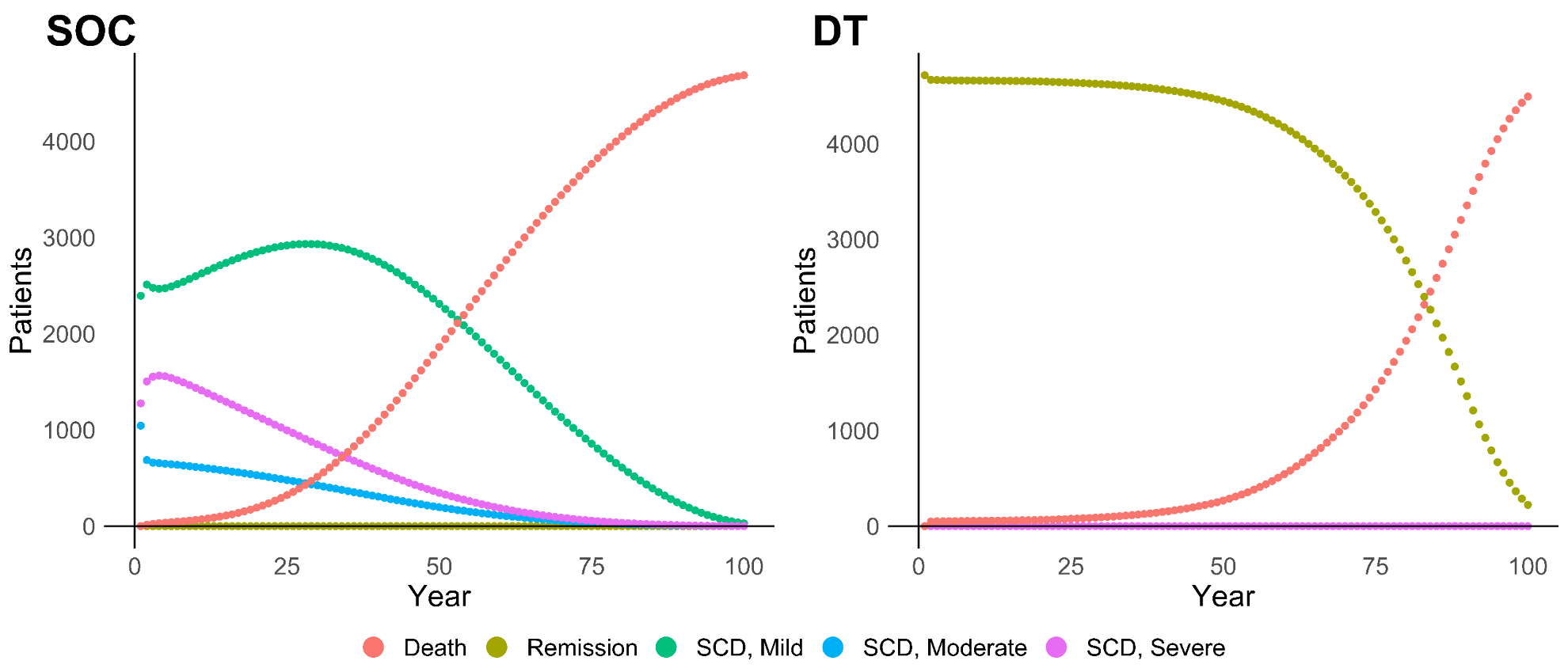
**

Abbreviations: SOC, standard of care; DT, durable therapy; SCD, sickle cell disease.

**Supplementary Figure 5** Markov trace for SOC and DT arms, males

**
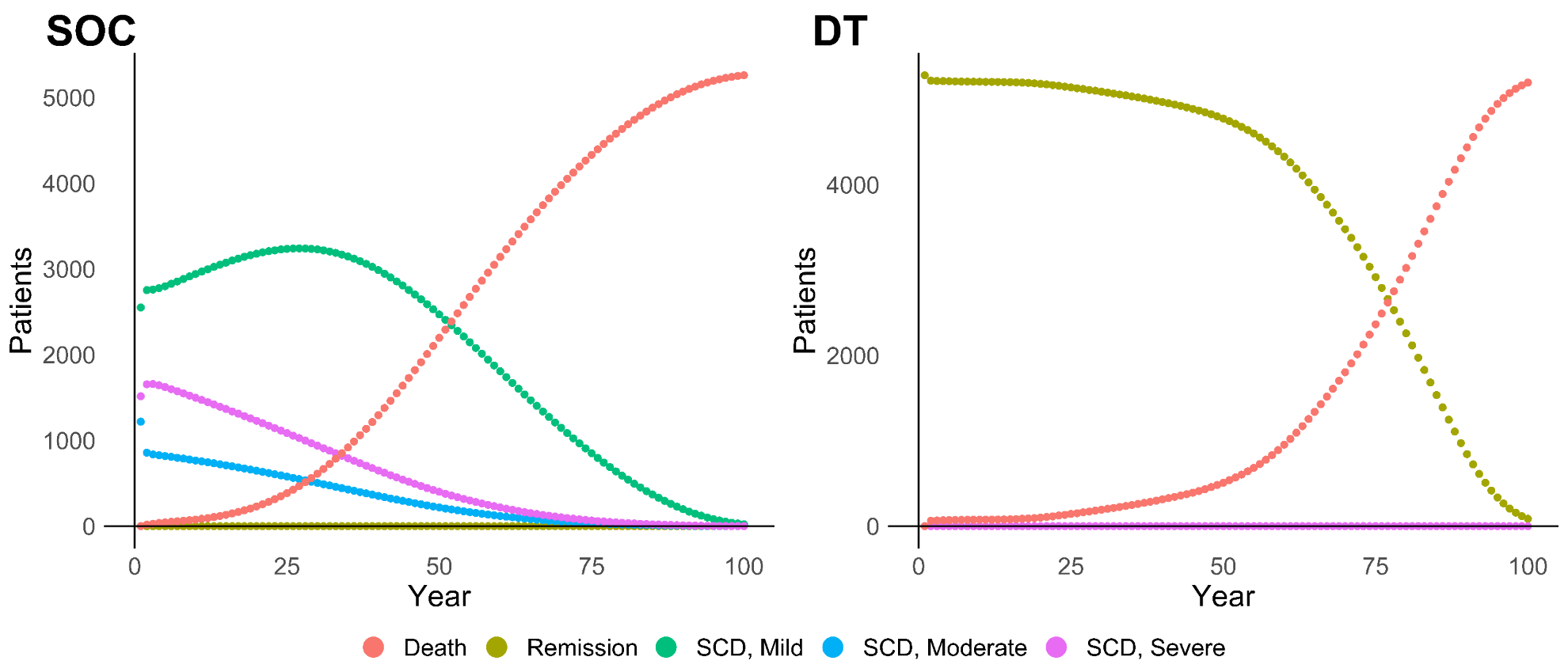
**

Abbreviations: SOC, standard of care; DT, durable therapy; SCD, sickle cell disease.

**Supplementary Figure 6** Monte Carlo draws (N=10,000), Cost parameters and coefficients for GLM log-link gamma family annualized total cost regressions

**
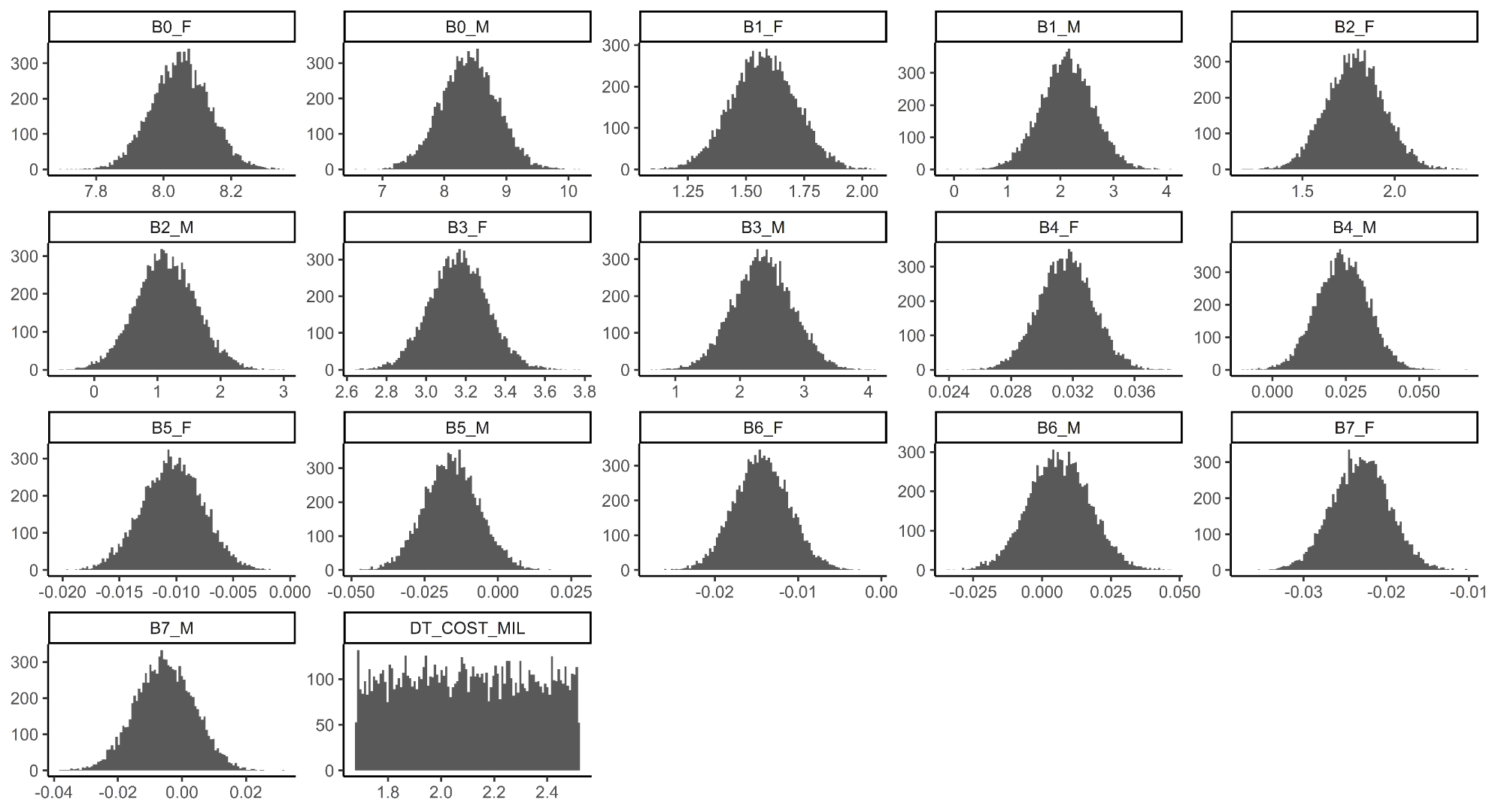
**

Abbreviations: GLM, generalized linear model; B, beta coefficient; M, males; F, females; DT, durable therapy; MIL, millions (2018 USD).

**Supplementary Figure 7** Monte Carlo draws (N=10,000), Utility parameters


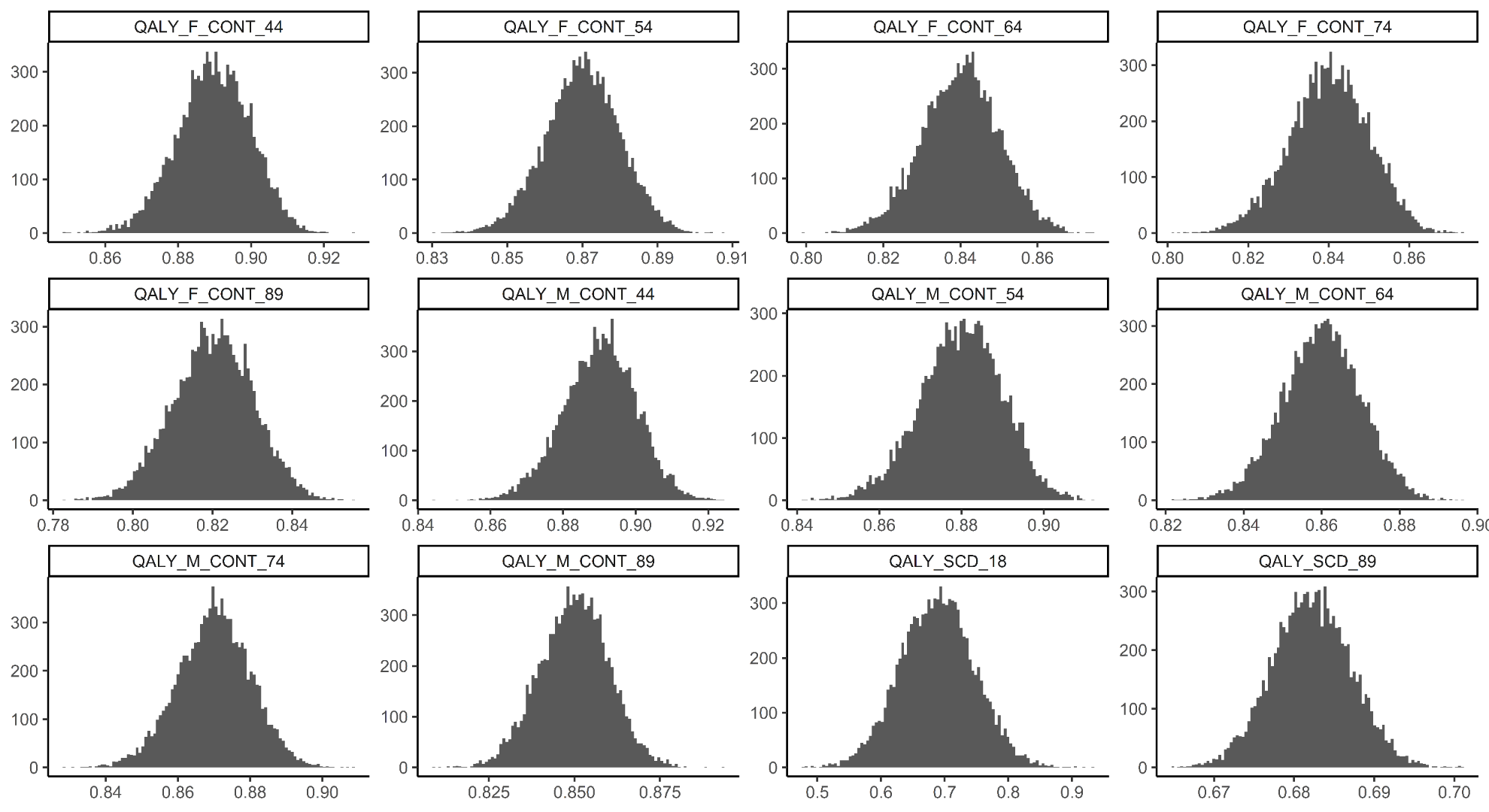


Abbreviations: QALY, quality-adjusted life year; F, females; CONT, control patients, M, males; SCD, sickle cell disease.

**Supplementary Figure 8** Monte Carlo draws (N=10,000), Initial condition and transition probability parameters: coefficients for ordered logit transition probability regressions.


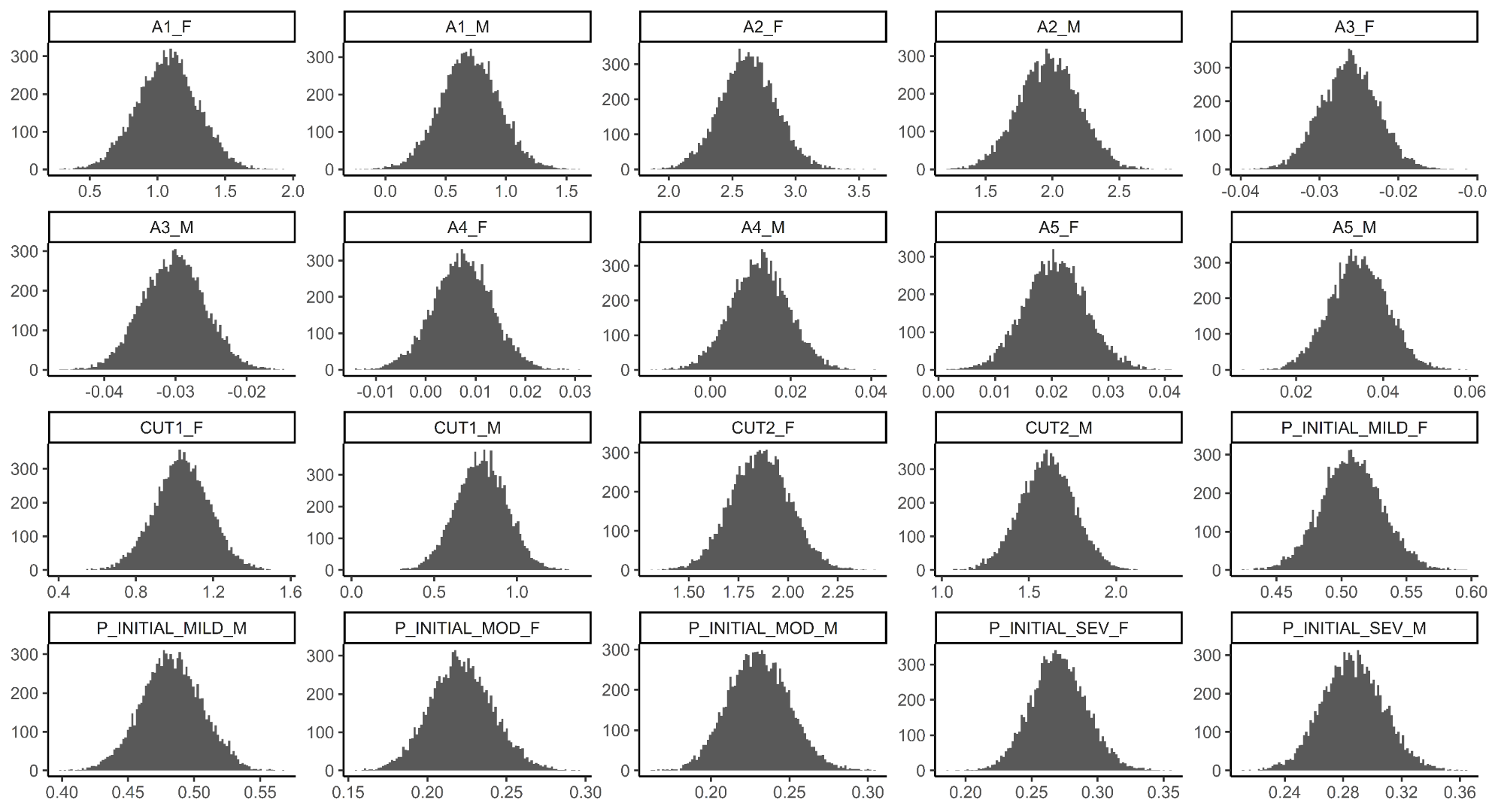


Abbreviations: A, alpha coefficient; F, females; M, males; mod, moderate; sev, severe.

**Supplementary Figure 9** Tornado diagram of deterministic sensitivity analysis results

**
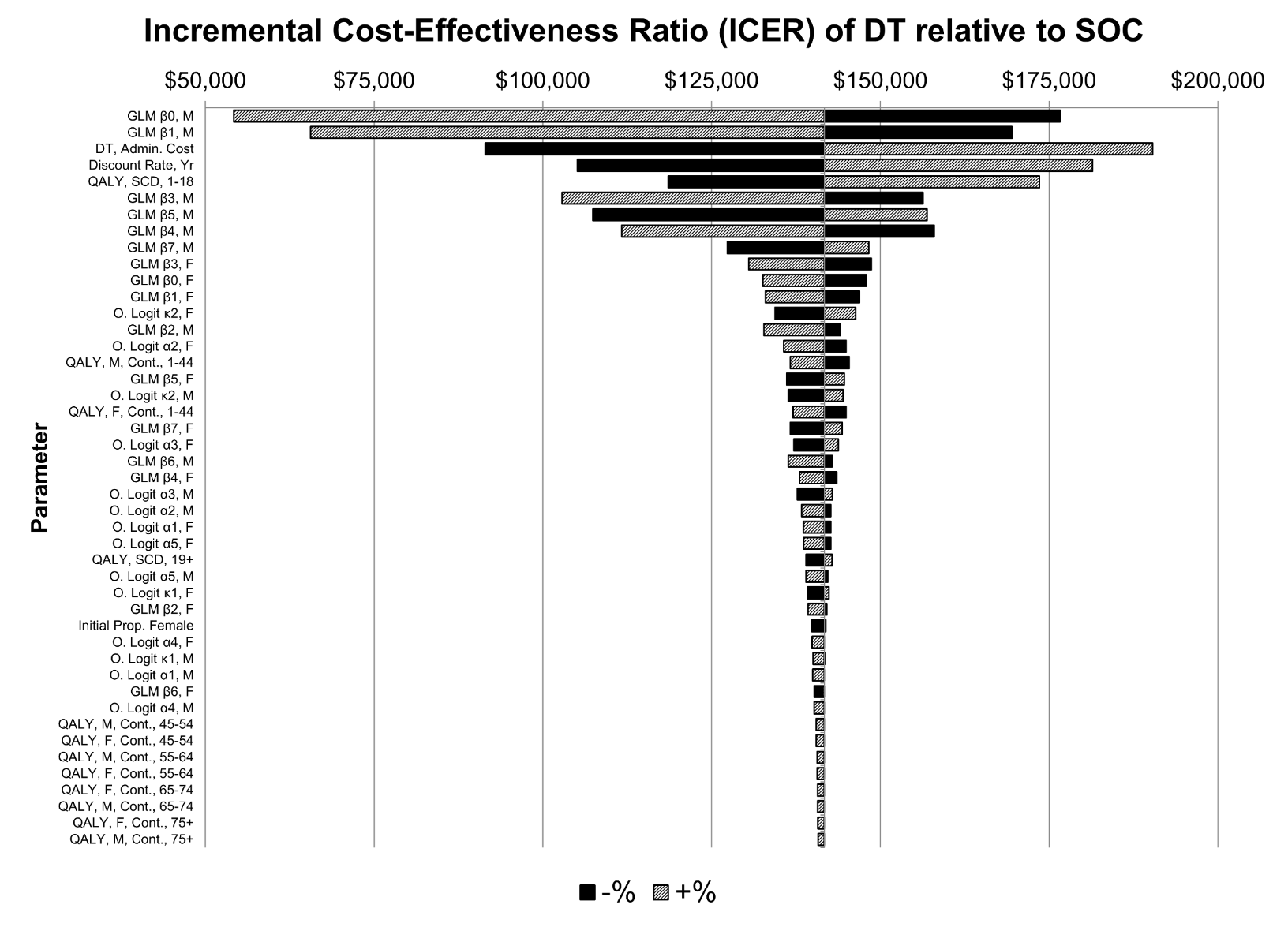
**

Parameters were varied univariately within their 95% CIs, or ± 20% when unavailable. Abbreviations: CI, confidence interval; SOC, standard of care; DT, durable therapy; SCD, sickle cell disease; Yr, year; QALY, quality-adjusted life year; GLM, generalized linear model; M, males; F, females; O. Logit, ordered logistic regression; Cont., control patients; Prop., proportion.

**Supplementary Figure 10** PSA results among females (A) Cost-effectiveness acceptability curves (CEAC) and frontier (CEAF); (B) Expected value of perfect information (EVPI)

**
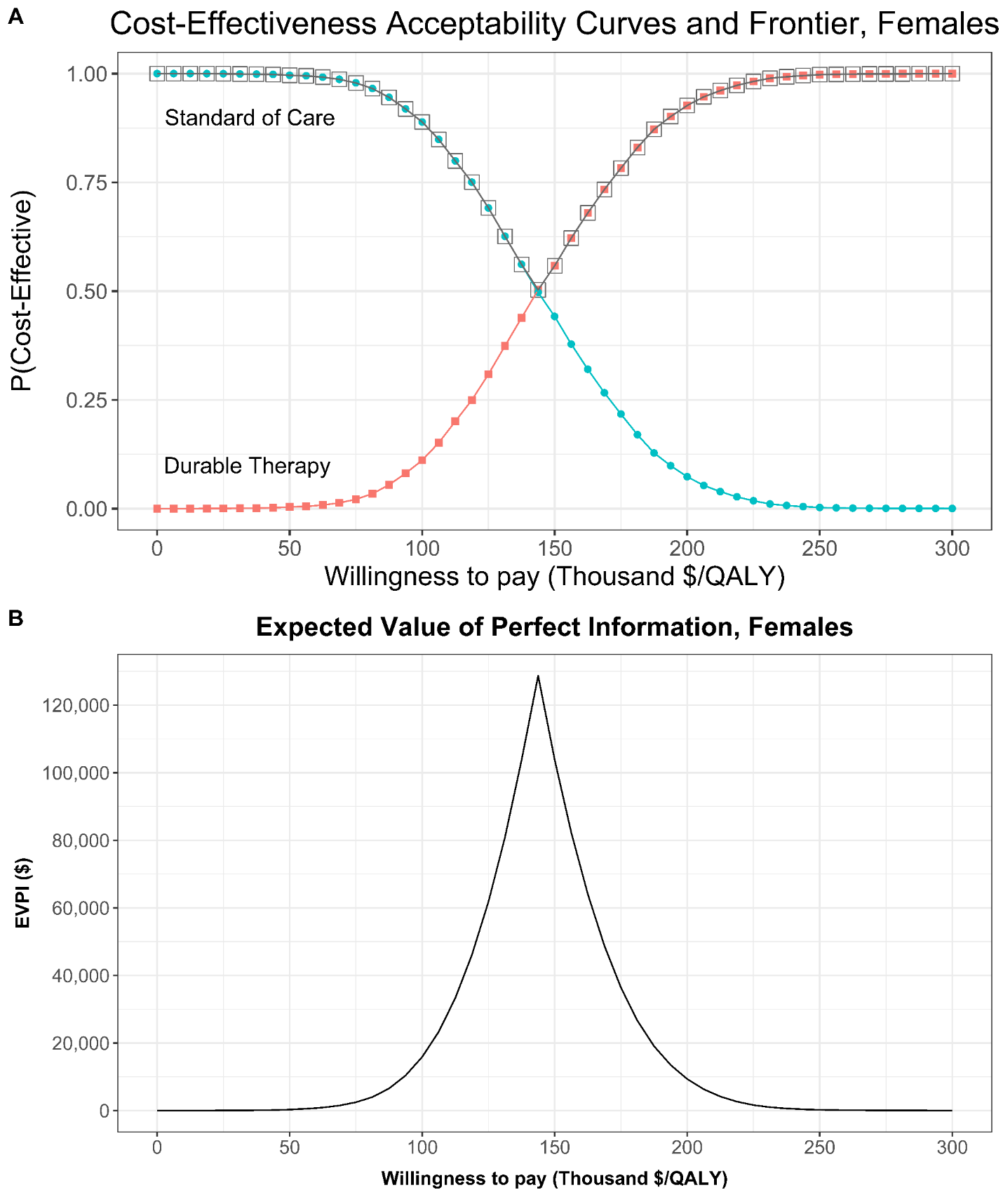
**

Abbreviations: PSA, probabilistic sensitivity analysis; QALY, quality-adjusted life year.

**Supplementary Figure 11** PSA results among males (A) Cost-effectiveness acceptability curves (CEAC) and frontier (CEAF); (B) Expected value of perfect information (EVPI)

**
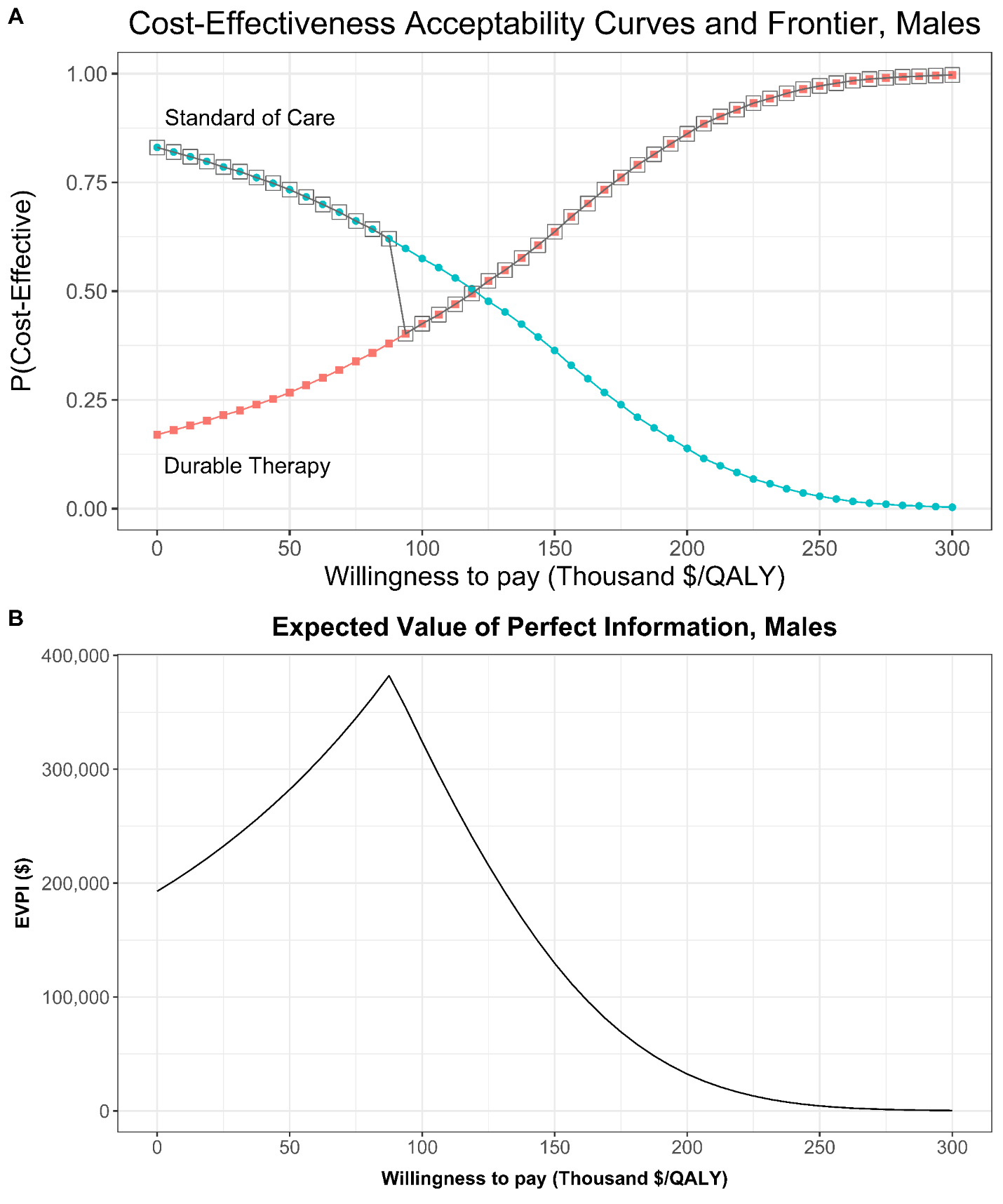
**

Abbreviations: PSA, probabilistic sensitivity analysis; QALY, quality-adjusted life year.
